# The NeuARt II system: a viewing tool for neuroanatomical data based on published neuroanatomical atlases

**DOI:** 10.1186/1471-2105-7-531

**Published:** 2006-12-13

**Authors:** Gully APC Burns, Wei-Cheng Cheng, Richard H Thompson, Larry W Swanson

**Affiliations:** 1 Information Sciences Institute, 4676 Admiralty Way, Marina Del Rey, CA 90292, USA; 2 Neuroscience Research Institute, Univeristy of Southern California, 3641 Watt Way, Los Angeles CA 90090-2520, USA

## Abstract

**Background:**

Anatomical studies of neural circuitry describing the basic wiring diagram of the brain produce intrinsically spatial, highly complex data of great value to the neuroscience community. Published neuroanatomical atlases provide a spatial framework for these studies. We have built an informatics framework based on these atlases for the representation of neuroanatomical knowledge. This framework not only captures current methods of anatomical data acquisition and analysis, it allows these studies to be collated, compared and synthesized within a single system.

**Results:**

We have developed an atlas-viewing application ('NeuARt II') in the Java language with unique functional properties. These include the ability to use copyrighted atlases as templates within which users may view, save and retrieve data-maps and annotate them with volumetric delineations. NeuARt II also permits users to view multiple levels on multiple atlases at once. Each data-map in this system is simply a stack of vector images with one image per atlas level, so any set of accurate drawings made onto a supported atlas (in vector graphics format) could be uploaded into NeuARt II. Presently the database is populated with a corpus of high-quality neuroanatomical data from the laboratory of Dr Larry Swanson (consisting 64 highly-detailed maps of PHAL tract-tracing experiments, made up of 1039 separate drawings that were published in 27 primary research publications over 17 years). Herein we take selective examples from these data to demonstrate the features of NeuArt II. Our informatics tool permits users to browse, query and compare these maps. The NeuARt II tool operates within a bioinformatics knowledge management platform (called 'NeuroScholar') either as a standalone or a plug-in application.

**Conclusion:**

Anatomical localization is fundamental to neuroscientific work and atlases provide an easily-understood framework that is widely used by neuroanatomists and non-neuroanatomists alike. NeuARt II, the neuroinformatics tool presented here, provides an accurate and powerful way of representing neuroanatomical data in the context of commonly-used brain atlases for visualization, comparison and analysis. Furthermore, it provides a framework that supports the delivery and manipulation of mapped data either as a standalone system or as a component in a larger knowledge management system.

## Background

Our objective within the current study was twofold: to construct informatics tools for neuroscientists to manage and use neuroanatomical knowledge within their everyday work and to provide support for neuroanatomical tract-tracing studies that elucidate the connections and chemoarchitecture of the brain. The entire design of our approach arose from practical methods from the laboratory of Professor Larry Swanson at the University of Southern California. The application we describe here is an extension, both technically and conceptually, of a prototype system originally called NeuARt [1,2].

The adage: 'all data are spatial' is especially pertinent in the field of neuroscience, since data is almost always indexed by the neuroanatomical location of phenomena or entities being studied. Previous attempts to construct atlas-based neuroanatomical databases have met with limited success due mainly to issues related to data registration across experiments. These issues are addressed implicitly by neuroanatomists when comparing nuclei and landmarks illustrated in a standard atlas to experimental data or published figures to one another.

Standard neuroanatomical atlases exist for many species such as the adult rat [3-8], the developing rat [9], the mouse [10,11], the hamster [12] and many others. They are typically large-format books (with supplemental electronic drawings on a CD) used in the day-to-day operation of a neuroscience laboratory. Thomson ISI's Web of Science lists a neuroanatomical atlas of the rat [13] as one of the most cited publications in science.

Generally speaking, an atlas is a series of sections from one or more brains of a given species, where all of the structures in each of the sections have been identified and delineated. They are usually published as a set of photographic plates and a corresponding set of drawings illustrating the delineated parts. In many cases the drawings are presented as separate figures, but delineations of brain structures may also be drawn or labeled directly on the photograph. Because every brain structure normally exists in all members of a given species in the same relative position, an individual brain with all parts outlined and labeled is a great aid to identifying the parts of an experimental brain. Other uses include finding stereotaxic coordinates for a defined position in brain tissue or using the atlas as a template to map data and facilitate comparisons across experiments.

Atlases provide two means of standardization: their spatial structure (*i.e*., where they place the borders of the individual structures) and their nomenclature. Atlases may also provide lists of synonyms within their indexes allowing some aspects of the complexity of neuroanatomical nomenclature found in published papers to be framed in a single set of named brain structures [5-7]. However, indexing structures only by name neglects spatial characteristics of the variations between different naming schemes.

Computational atlases feature prominently in existing bioinformatics projects. One example of this is the Allen Reference Atlas [14] as part of the Allen Brain Atlas project. The ambitious goal of this project is to map every single gene in the mouse brain onto an online atlas using high-throughput *in-situ *hybridization techniques. The BrainInfo project [15,16] has an online template atlas for the Macaque, which has also been published commercially [17]. The BrainMaps.org website presents photographic plates of macaque and mouse brains with a browser that permits zooming into the data images [18,19]. The Mouse Brain Library provides online access to a mouse atlas [20]. The Mouse Atlas Project (MAP) at the Laboratory of Neural Imaging (LONI) at UCLA has linked together block-face imaging, high-resolution magnetic resonance imaging and histological imaging in a single 'multimedia' atlas [21,22]. The SmartAtlas project of the Brain Information Research Network (BIRN) at UC San Diego is based on published atlases and also provides GIS-based spatial indexing functionality [23,24]. The Neural Systems and Graphics Computing Laboratory at the University of Oslo in Norway, build highly precise representations of specific systems as well as general neuroinformatics tools concerned with rodent neuroanatomy [25,26].

One goal of these projects is often to deliver atlas material online. Whether on the web, or in any other form, published atlases are protected by copyright laws and may not be distributed without appropriate licensing. This is a problem of particular relevance for developers of web-applications, since publishers are reluctant to provide permission for free open-access usage of atlas plates.

Before describing the application itself, which solves the copyright problem, it may be helpful to give a brief overview of neuroanatomical methods of data collection. In general terms, current tract-tracing experiments involve a small quantity of tracer, deposited into a specified region of the brain. Within this 'injection site', the tracer is taken up by neurons and/or their processes (axons, dendrites) and actively transported either 'anterogradely' (away from the cell body) or 'retrogradely' (towards the cell body). Anterograde tract tracers such as *Phaseolus vulgaris *leucoagglutinin (PHAL) generally fill the entire length of the axon including its terminal boutons. Retrograde tracers (*e.g*., Fluorogold [49]) are taken up predominantly by axon terminals and fill their neurons of origin. In either case, the tracer is selectively visualized with standard methodologies (such as enzyme- or fluorescence-based immunohistochemistry).

By comparing the pattern of projections from adjacent or overlapping injections it is possible to differentiate cell groups (gray matter regions) based on their extrinsic outputs and inputs. Thus, the predominant definition of a brain region (nucleus, area, or cell group) is 'a brain locus containing a population of neurons that give rise to a characteristic and distinguishable set of extrinsic inputs and outputs'. In many cases, the definitions of individual regions can be refined further based on gradients in the projections or regional differences in the expression of certain molecules such as peptides, proteins, or neurotransmitters. Because the projections mediate the function of that group of neurons, tract-tracing experiments seek to describe the connections of the brain to identify circuits that constitute the component systems that subserve brain function.

**Figure 1:**
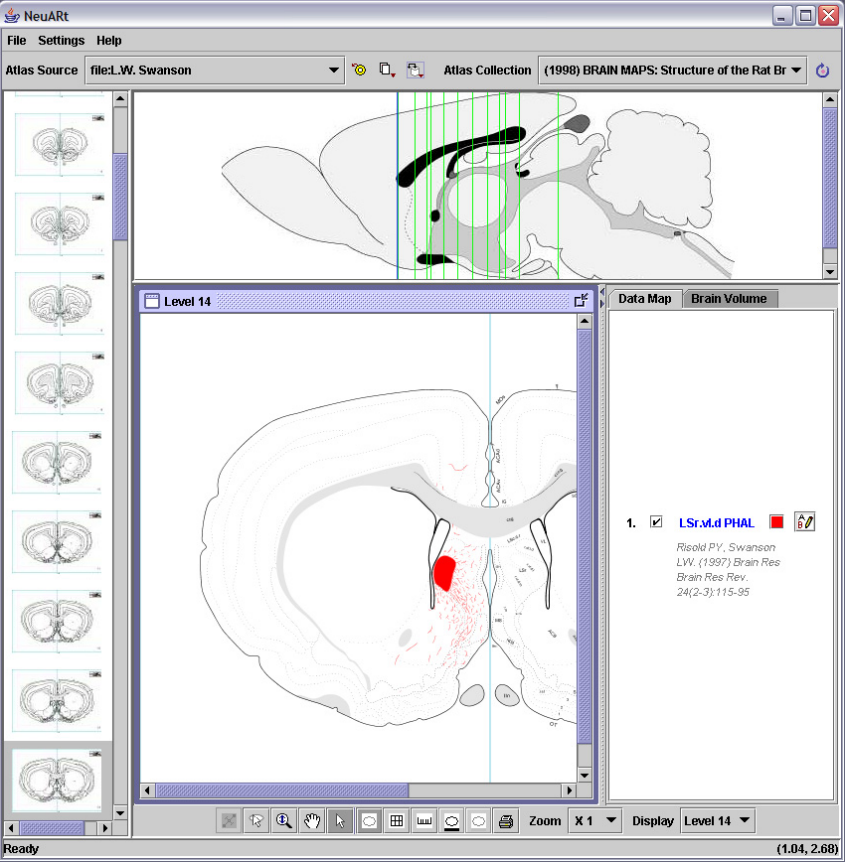
Screenshot of the NeuARt II system showing the simplest view available for a single case across a single level of a PHAL injection into the rostral part of the lateral septal nucleus, ventrolateral zone, dorsal region (LSr.vl.d). Reprinted from Brain Research Reviews, vol 24, Risold, P.Y. and L.W. Swanson "Connections of the rat lateral septal complex". pp115-95, Copyright (1997), with permission from Elsevier.

Figure 1 shows the basic view of the NeuARt II application for data from a typical tract-tracing experiment. In this example, PHAL was injected into a subregion of the lateral septal nucleus, see [27] (see the Results section below). In a single view, the application presents an atlas level with the data registered in a superimposed layer. Here, the data consists of both the injection site (as a filled shape) and the labeled fibers/axons (as spline curves). The application interface also includes an idealized midsagittal (or 'longitudinal') section for showing the position of all atlas levels for which data has been mapped in this experiment. In the far left-hand panel of the application, there is a thumbnail series of coronal (cross-sectional or transverse) sections through the brain that represents all levels of the current atlas.

**Figure 2:**
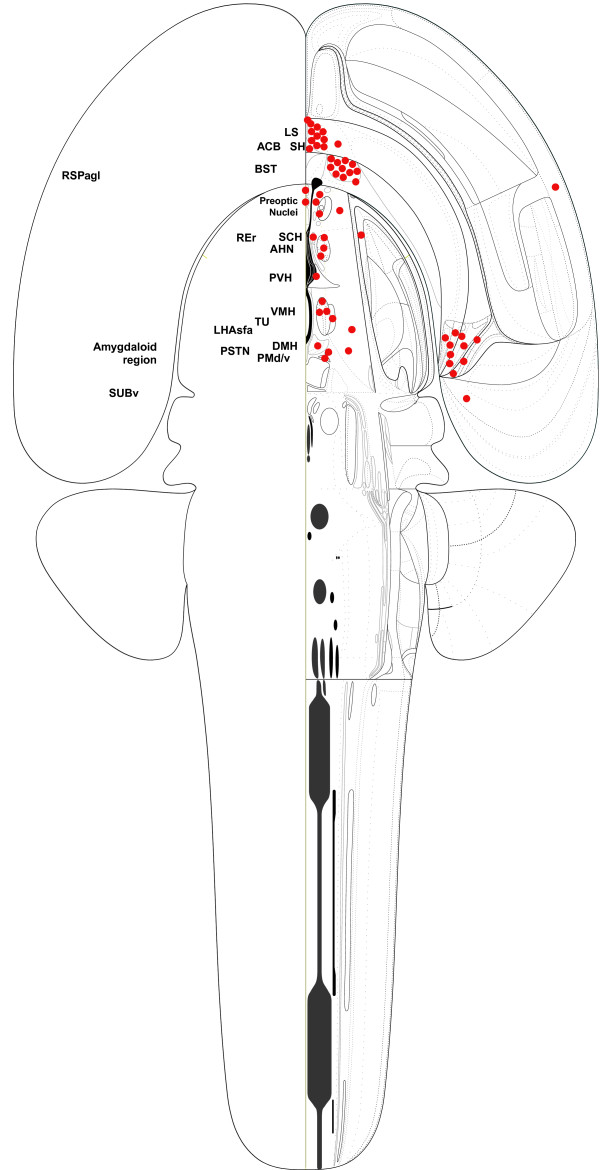
Injection sites of complete PHAL tract-tracing experiments published by the Swanson laboratory 1989–2006 mapped onto a 'flat-map' of the rat brain. Within this diagrammatic construct, the position and relative size of each region is based on a 'fate-map' of its region of origin on the neural plate (itself a two dimensional structure; see [50, 51]). At the time of writing, NeuARt II contains 64 data-maps consisting of PHAL experiments (n = 56), immunohistochemical maps (n = 5), Fluoro-Gold tract-tracing experiments (n = 1), and in-situ hybridization maps (n = 2). Within this paper, we provide a table of abbreviations for those mentions from the text and in Figures. The nomenclature presented here is that of [6].

Although NeuArt II currently accepts data mapped onto either of two major rat atlases and efforts are underway to expand the range of data included, at this point it is largely populated by a single source. For more than twenty years, the Swanson laboratory has produced detailed descriptions, including high resolution maps, of the connections of the rat brain, concentrating primarily on the hypothalamus, amygdala, and septal complex, amongst others [27-51]. The majority of these studies used PHAL [52], whilst others variously using retrograde tracing, immunohistochemistry, and *in-situ *hybridization histochemical techniques. Figure 2 shows a two-dimensional schematic diagram of the entire adult rat brain summarizing the injection sites of the experiments currently available within NeurArt II.

Each of these studies consists of one or more data sets describing the projections of a given region (plus the appropriate controls). Each data set consists of a representation of the injection site and as many drawings as necessary to describe the axonal projections of the region under study. All drawings (including the ones that contain the injection site) are made, or mapped, onto an atlas template from [6]. We use the term 'data-map' to denote 'a set of drawings across atlas levels that collectively describes data from a single experiment'.

Note also that this form of representation inherently produces results that are registered into the same spatial framework. This allows drawings from different experiments to be mapped onto a single atlas level and then visually searched for patterns of overlap and separation. While this method of representing anatomical data differs fundamentally from more literal methods such as camera-lucida drawing (see Discussion, below), we have argued that, when performed by a trained neuroanatomist, this method produces accurate and precise maps that facilitate qualitative comparisons across experiments [5-7]. In addition the method lends itself readily to more formal, computational approaches.

Our software, NeuARt II, improves upon the current method by providing tools that facilitate data comparison within and across experiments. While the database is currently populated with data from a single source, the principle applies to any commercially available atlas and has been implemented to support the two major atlases of the rat brain [6,8]. Moreover, this system is still under active development. It will be able to fully implement spatial indexing and structured meta-data. Its current ability to operate within the NeuroScholar bioinformatics knowledge management platform means that it currently supports data consolidation across communities. Preliminary results have been published in poster format at the 2005 Society for Neuroscience annual meeting [53].

## Implementation

The system is developed in Java 1.4.2 and is stable on Windows XP and Mac OS X (v10.1–10.3). The system has also been implemented on the current Mac OS 10.4 ('Tiger'), but conflicts between the OS and the Batik SVG library cause the program to run slowly. In the future, we will support the process of developing a solution that works reliably on Apple machines.

Complete source code for NeuARt II is available from the project page on SourceForge [54] and the program is deployed as an installer package, see the NeuroScholar website [55] for more documentation about downloading and installing the system. Because NeuARt II requires an underlying installation of the MySQL database (which may be packaged with the installer if required), setting up the database requires some previous experience administering MySQL systems. Imported files are converted to the SVG format ('Standard Vector Graphics', [56]). This format was adopted for its high-quality open source graphics library (Batik; [57]). To use the system and preserve copyright, the user must own a copy of the appropriate atlas and Adobe Illustrator (v.10 or higher). The system currently supports Swanson's 2^nd ^and 3^rd ^edition atlases [6,7] and Paxinos 5^th ^edition [8] with additions to follow. To supply as much useful material for developers of other systems, we describe our underlying database schema (see Appendix A) and refer readers to the NeuARt II Developers Manual for an extensive description of the systems' application programming interface ('API') [58].

### Basic Usage and Data Flow

**Figure 3:**
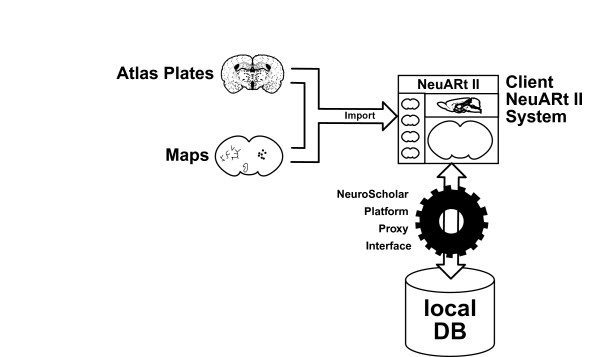
Overall system component architecture and data flow of the NeuARt II system. Interaction under the NeuroScholar 'proxy' mechanism involves converting the internal java-based data representation from within the NeuARt II interface to the NeuroScholar knowledge representation under the View-Primitive-Data-Model-framework ('VPDMf') [59]. The NeuroScholar platform's API may then operate directly on this representation.

Figure 3 illustrates data flow between the system's external sources of data (*i.e*., atlas plates and neuroanatomical data mapped onto atlas plates), the user interface and the system's local database (implemented via the persistence mechanism of the NeuroScholar platform [59]). The use of this software is based on published neuroanatomical atlases, which are themselves commercial products commonly found in neuroscience laboratories. The user must own a copy of the atlas, in order to preserve the copyright of the atlas' publishers. Furthermore, all currently supported atlases [6,7] provide electronic versions of their atlas plates as Adobe Illustrator files and thus Adobe Illustrator, as a commercial product, must also be purchased to use standard atlases within NeuARt II. After recognition of a valid atlas CD, NeuARt II launches a Javascript program, run within Illustrator, to convert the atlas files into the SVG format and copy them to the local system. This process need only be performed once for each atlas. In this way we are able to provide open-source, freely available software that capitalizes upon and extends the functionality of commercially available software (neuroanatomical atlases), while avoiding major copyright issues. The NeuARt II system accommodates less stringent usage conditions, so it can accommodate open-source atlases if they become available in the future.

Conceptually similar to the support for multiple atlases is support for multiple editions of a given atlas. New editions of an atlas may involve updates in anatomical delineations and nomenclature, but when they based on the same brain (so that the overall shape and number of levels are unchanged) the registration across editions is preserved. Under these conditions, NeuARt II permits the user to compare visually the delineations of different atlas editions.

In this regard, Elsevier Science (Amsterdam) has given permission for the use of Larry Swanson's 2nd and 3rd edition atlases [6,7], and George Paxinos' 5th edition atlas [8] with the copyright restrictions noted above. At present, NeuARt requires that the user select one primary atlas, but allows any co-registered atlas to be superimposed over the first. However, formal methods of co-registration between atlases are not yet provided. Published atlases were chosen as our starting point because, in effect, any data mapped onto a published atlas is in registration with all other studies mapped onto that atlas. Furthermore, the approach to integrating data across atlases is to provide visual displays that permit side-by-side (and layer-by-layer) comparisons rather than directly transforming data sets algorithmically. Note that data mapped onto a given atlas is specific only to that atlas. The difficult problem of mathematically translating data from one histological atlas to another (or one histologically sectioned brain to another) has not yet been solved adequately in this or any other system. Within this system, the action of editing data maps is currently performed by editing the original Adobe Illustrator data files and uploading those files to overwrite existing data. Although not the ideal solution to the problem, this approach reflects existing methods of comparing neuroanatomical maps and avoids problems associated with warping and other transformations (at least until accurate and standardized methods become available).

The process of uploading data-maps to NeuARt II is accomplished with the use of an automated 'wizard' (a dialog box guiding users through a multi-stage input process). For a data set with drawings on a set of atlas levels, the user must register each drawing on each of the atlas plates referenced by the data set and save the data as a set of SVG files (one for each atlas level). The files must be in accordance to a naming template that requires the level number of the atlas to be consistently present in the file name (*e.g*., 'data-XX.svg' where XX has a numerical range over the number of atlas levels and takes the same value as the atlas level to which it refers). Once the data has been loaded, it may be saved to the underlying system's database. For the purposes of demonstrating this functionality practically, we provide a sample dataset as an additional file (Additional file 1: sample.zip).

In theory, NeuARt II can support any atlas that provides images of its plates as Adobe Illustrator files (or any vector graphics format that may be converted to SVG format). Within the current java source code, all program files that are required to build an interface for any given atlas can be found in the 'edu.usc.kmrg.tools.atlasMapper.brainmapImport' package. This contains code templates for existing scripts that may be adapted by developers wishing to extend NeuARt's functionality.

### Design of the Knowledge Representation

**Figure 4:**
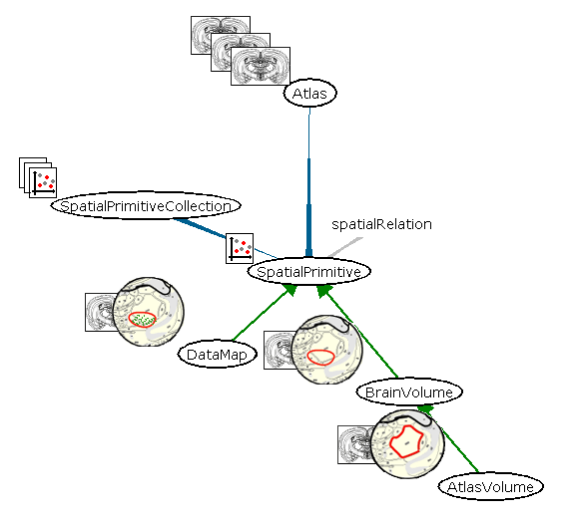
The data schema of the NeuARt II application, shown as a partial screenshot taken from the NeuroScholar browser. NeuroScholar and NeuARt II use the same underlying knowledge management core system (called the View-Primitive-Data-Model-framework or 'VPDMf', [59]).

Data persistence in NeuARt II is handled under the NeuroScholar knowledge management platform [60-64]. The NeuroScholar project is an open-source, research prototype that uses the MySQL open-source relational database and can therefore scale to contain hundreds of thousands of data instances. Figure 4 shows the schema for spatial objects used within the system.

Within the NeuroScholar browser itself each node is an interactive object that may be queried within the system's browser-like user interface [63]. As is shown in Figure 4, the schema for NeuArt II is centered on the 'spatial-primitive' node (which cannot itself be instantiated). Every spatial-primitive links to an atlas, and may have spatial relations (such as overlap, inclusion, identity, *etc*.) with other spatial-primitive objects (see [65] for automated reasoning approaches based on these relations). They also may be part of a spatial primitive collection. We use three subtypes of spatial-primitive: data-maps, brain-volumes and atlas-volumes.

Data-maps are defined as drawings overlaid onto, and in register with, the atlas plates. These drawings may have any graphical content and the function of the NeuARt II system is to register and render these drawings appropriately. Additionally, we have created a construct called a 'brain-volume', which is a set of closed spline curves placed onto consecutive atlas plates and together constitute a volumetric delineation within the framework of the atlas. Each spline control point has an adjustable 'width' that is designed to act as an 'error bar' to describe uncertainty in the delineation.

Brain volumes are currently defined by a single surface, *i.e*., they cannot contain holes. Adding, editing and deleting brain-volumes are all managed from within the NeuARt II interface. Atlas-volume views are simply the named regions within the atlas itself. In this, the first practical release of the system, metadata for individual data-maps is currently provided only by 'name' and 'description' fields where free text may be entered to describe the features of the data set. More detailed semantic meta-data (such as location of injection sites and labeled regions) is implemented from within the NeuroScholar system [66]. Consistent with this, the user may query the system via a simple interface with four fields: a pulldown combo-box for 'Type' of spatial primitive with the options 'DataMap', 'BrainVolume' or 'AtlasVolume'; text boxes for the 'Name' and 'Description' of the spatial-primitive and another pulldown combo-box for 'Atlas' (referring to each atlas in the system). These queries return a list of instances of the three types of spatial primitive in the system. The user may then select one to be viewed in the NeuARt II interface.

## Results

The NeuArt II system has several sets of features. These are (A) the incorporation of standard published atlases and data with no violation of copyright (as described above); (B) a variety of viewing functions such as pan, zoom, stereotaxic coordinate navigation and rulers; (C) the ability to view multiple atlas plates simultaneously in multiple windows (each with the capability of overlaying multiple data sets in independent layers); (D) data storage and retrieval functions involving 'data-maps', (E) the capability of delineating 'brain-volumes'; and (F) integrated functionality with the NeuroScholar knowledge management platform. In this section, we describe these features in detail with examples taken from the Swanson laboratory corpus of tract-tracing and chemoarchitectonic data [27,45].

**Figure 5:**
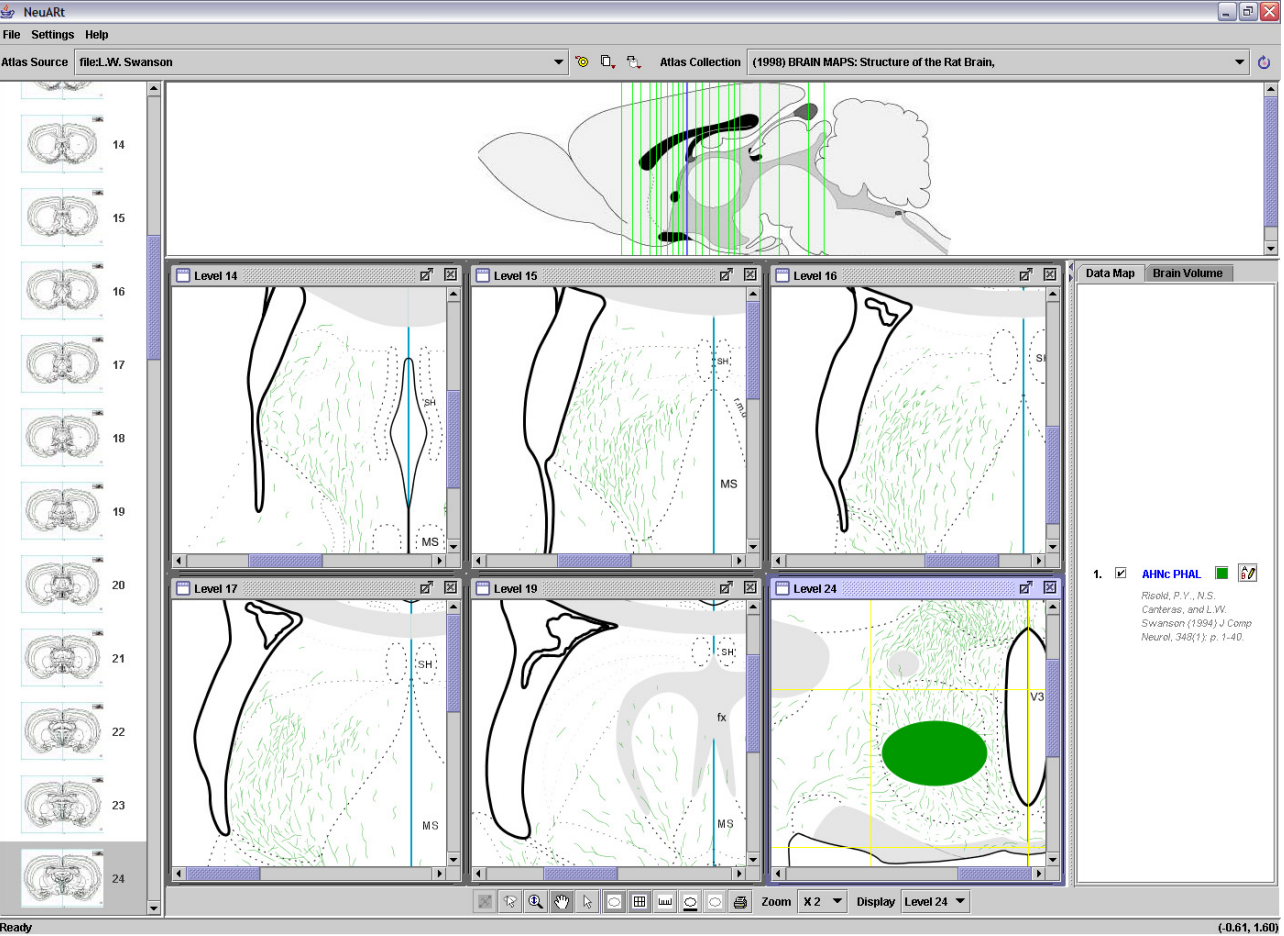
Screenshot of NeuARt II illustrating the full extent of a single case across multiple levels for a PHAL injection into the central part of the Anterior Hypothalamic Nucleus (AHNc). Reproduced with permission of John Wiley & Sons, Inc. from Journal of Comparative Neurology, vol 348, Risold, P.Y. and L.W. Swanson "Organization of projections from the anterior hypothalamic nucleus: a Phaseolus vulgaris-leucoagglutinin study in the rat". Pages 1–40, Copyright ^© ^1994 Wiley Periodicals, Inc.

A more detailed view of the NeuARt II system, illustrating multiple drawings from a single experiment, is shown as a screenshot in Figure 5. In the description below, we list the key features of the system and their usage. Note that while the system's core design have been firmly established, we expect details to continue to evolve as we implement upgrades and respond to feedback from users.

In this release, the application is split into three panels: a list of thumbnails of the atlas' coronal (transverse) plates on the left, a sagittal (longitudinal) view at the top and the main panel on the right with a list of data-maps that are currently being viewed (Fig. 5). The thumbnails and sagittal views are provided for navigation purposes. Within the sagittal view, the vertical lines indicate the number and atlas level of maps in the data set. The main panel has a single active subpanel, for which additional functions become available via buttons located at the bottom of the screen. These support zooming, panning, selection, scale rulers (not shown), and a grid tool that superimposes a stereotaxic grid over the atlas images. The grid and ruler tools provide convenient points of reference when aligning multiple maps to compare data across levels. For precise measurements the physical coordinates of the pointer position are displayed in the bottom right corner and are continuously updated as the mouse is moved.

The anatomical drawings in Figure 5 were taken from a data-map of an experiment (AHN-22) within a single study of the projections of the anterior hypothalamic nucleus (AHN; see Figure 4 in [44]). Here we have selected drawings of AHN projections, emphasizing those to the lateral septal nucleus and illustrating the injection site (as a subset of the drawings available). The range and total number of drawings available in this data-map is indicated by the lines in the sagittal view.

Users may simultaneously view multiple atlas plates (with overlaid data) by right-clicking the coronal thumbnails to the left. This permits users to make comparisons across plates in the rostrocaudal (Z) direction. Neuroanatomists generally map the brain at a sampling frequency high enough to represent the essential features of any data set. In part because most neural structures span more than one atlas level, (and in part because of the time and effort involved in producing each map) this rarely involves all, or even adjacent, atlas levels. This may mean that only neighboring atlas levels are available when comparing two experiments.

**Figure 6:**
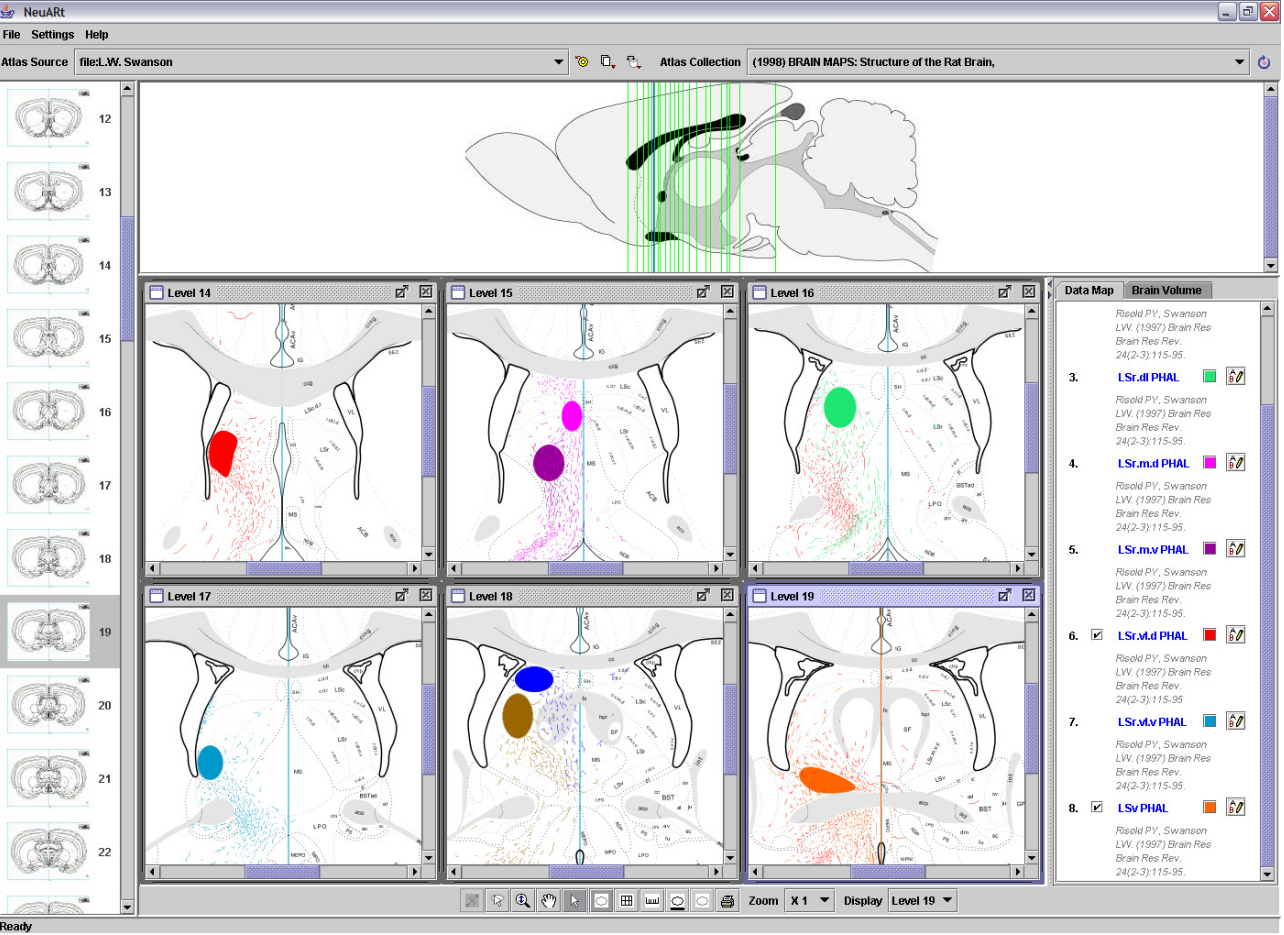
Screenshot of NeuARt II illustrating the injection sites from a set of PHAL tract-tracing experiments examining the efferent connectivity of sub-compartments of the lateral septal complex. Reprinted from Brain Research Reviews, vol 24, Risold, P.Y. and L.W. Swanson "Connections of the rat lateral septal complex". pp115-95, Copyright (1997), with permission from Elsevier.

Figure 6 illustrates how the system may be used to compare injection sites within and across anatomical levels. All injection sites are experiments taken from a study of the projections of the lateral septal complex [41]. Because these experiments are in register, the location of each injection site with respect to the atlas and relative to each of the other injections can be compared. Having multiple windows with the relevant atlas plates allows us to see directly that these are non-overlapping injections distributed broadly within the lateral septal nucleus.

The number of open windows is limited primarily by the size of the screen. We have tested the system with ten windows open simultaneously and the system still has good response- and redraw-times. Although this cannot be shown in static images, the user is able to dynamically toggle the visibility of each map independently, change the order of the layering (with those higher in the list masking lower maps) and change the color of the data map and its constituent parts. These features are powerful tools that enable users to compare data-maps within and across atlas levels and to rapidly identify meaningful patterns even as the number of layers increases.

**Figure 7:**
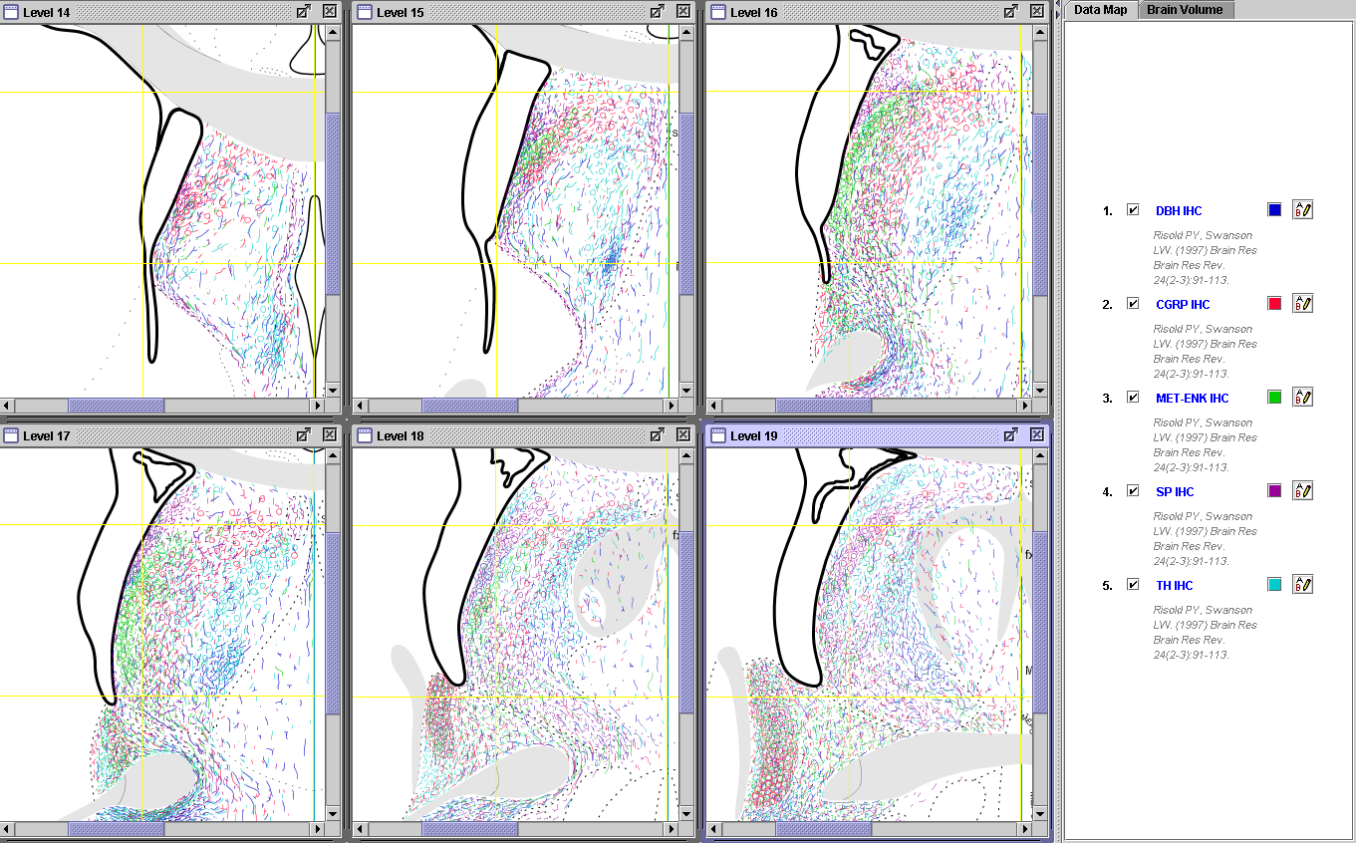
Screenshot of NeuARt II illustrating the use of NeuARt II to view non-tract-tracing data, in this case, immunohistochemical labeling of enkephalin, calcitonin gene-related peptide (CGRP), substance P (SP), dopamine β-hydroxylase (DBH), and tyrosine hydroxylase (TH) within the different parts of the lateral septal nucleus (see Figure 8 from [45]). Maps reprinted from Brain Research Reviews, vol 24, Risold, P.Y. and L.W. Swanson "Chemoarchitecture of the rat lateral septal complex". pp91-113, Copyright (1997), with permission from Elsevier.

Another level of organization frequently studied by neuroanatomists is the distribution of chemically identified neurons and their axons. Neurons differentially express a wide variety of substances (*e.g*., neurotransmitters, peptides and their synthetic enzymes, *etc*.). Examples of this type of study, and its associated maps, are shown in Figure 7 where the distribution of labeled fibers (lines) and pericellular baskets (large specializations of an axon terminal that appear to surround the neuronal cell body, shown as open circles) have been drawn for three peptides (enkephalin, calcitonin gene-related peptide or 'CGRP', and Substance P or 'SP') and two neurotransmitter synthetic enzymes (dopamine β-hydroxylase or 'DBH', and tyrosine hydroxylase or 'TH') within the lateral septal nucleus. The fact that a group of neurons expresses a particular molecule, or that a region receives a characteristic input of a particular type may be taken as evidence of the intrinsic connectional organization of parts or systems in the brain. In some instances histochemical labeling coincides with cytoarchitecturally-defined borders, however, it is often the case that the pattern of labeling crosses boundaries, offering an alternative method for organizing brain structure. This approach is generally called 'chemoarchitectonics'. Generally speaking, the method of data representation is similar to tract tracing studies. Perhaps more importantly, all of the the histochemistry data-maps in Figure 7 are in register with previous tract tracing data-maps because they were drawn onto an atlas template. Thus atlas-based maps provide a robust method to visualize all major classes of neuroanatomical data, allowing precise comparisons across experiments in a dynamic interface.

**Figure 8:**
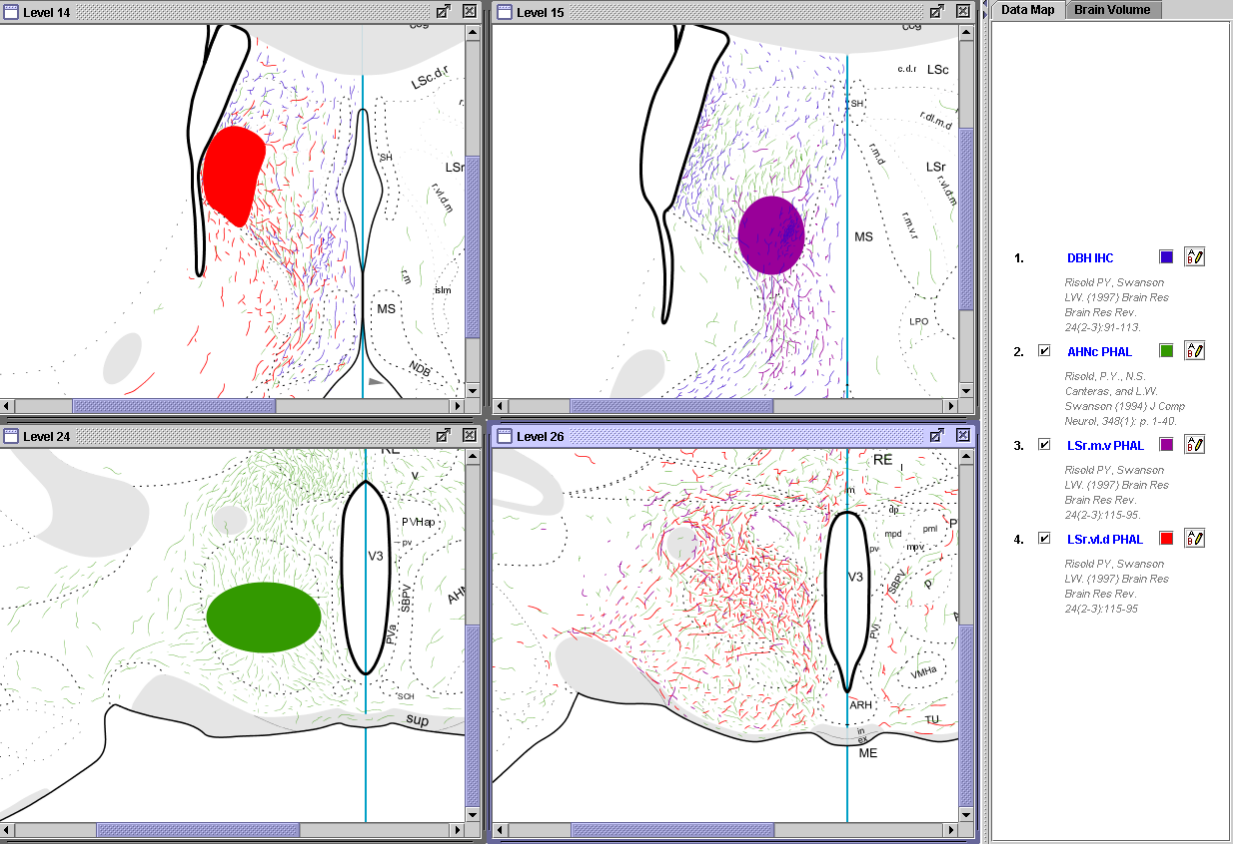
Screenshot of the NeuARt system showing two levels of comparison of inputs and outputs of the lateral septal nucleus. Levels 14 and 15 show how the projections of a set of inputs (both identified afferents and endogenous molecules) can be inferred. In these two panels, note that PHAL injection sites (in LSr.vl.d (red) and LSr.m.v (black), respectively) overlap with axons immunostained for DBH (blue) and green afferent fibers from the PHAL injection in the AHN Level 24. Level 26 shows that the projections from some DBH- and AHN-recipient parts of the LS converge with each other as well as with the projections from the AHN in at least one level of the hypothalamus. Thus it is possible to use the NeuARt system to compare multiple data-sets arising from different classes of experiment, to illustrate novel results and generate testable hypotheses about the brain's circuitry. Maps reprinted from Brain Research Reviews, vol 24, Risold, P.Y. and L.W. Swanson "Chemoarchitecture of the rat lateral septal complex". pp91-113, Copyright (1997), with permission from Elsevier. Reprinted from Brain Research Reviews, vol 24, Risold, P.Y. and L.W. Swanson "Connections of the rat lateral septal complex". pp115-95, Copyright (1997), with permission from Elsevier. Reproduced with permission of John Wiley & Sons, Inc. from Journal of Comparative Neurology, vol 348, Risold, P.Y. and L.W. Swanson "Organization of projections from the anterior hypothalamic nucleus: a Phaseolus vulgaris-leucoagglutinin study in the rat". Pages 1–40, Copyright ^© ^1994 Wiley Periodicals, Inc.

The combination of histochemistry and tract tracing is referred to as 'chemical neuroanatomy.' It also is common practice to use immunohistochemistry as a marker to provide points of reference to visualize anatomical borders or other features. Notice that there are several regions in the LS where projections from the AHN (Fig. 5) overlap (and even resemble) the immunohistochemical labeling (Fig. 7). By comparing the fiber labeling with locations of the PHAL injections (Fig. 6) we can begin asking questions about the pattern of projections generated by the region that receives this convergent input. Figure 8(A–C) shows the relevant parts of each data set needed to address this question: the coincident distribution of DBH-labeled fibers and PHAL-labeled fibers from the AHN (Fig. 8A and 8B); the location of the PHAL injection in the AHN that gives rise to the LS input; and two PHAL injection sites centered in DBH/AHN-recipient regions of the LS. An example of the projections from these two injection sites are shown in Figure 8D. The distribution of fibers in this map suggests that these two regions of the LS that receive the combined DBH/AHN input in turn project back to the AHN, among other places. In this way, we are able to integrate, visually compare, and synthesize data from three separate publications [44,45,27] in a spatially consistent manner. These comparisons then suggest a novel result, allowing us to make inferences about the organization of brain circuitry. This example illustrates the usage and potential power of this approach.

**Figure 9:**
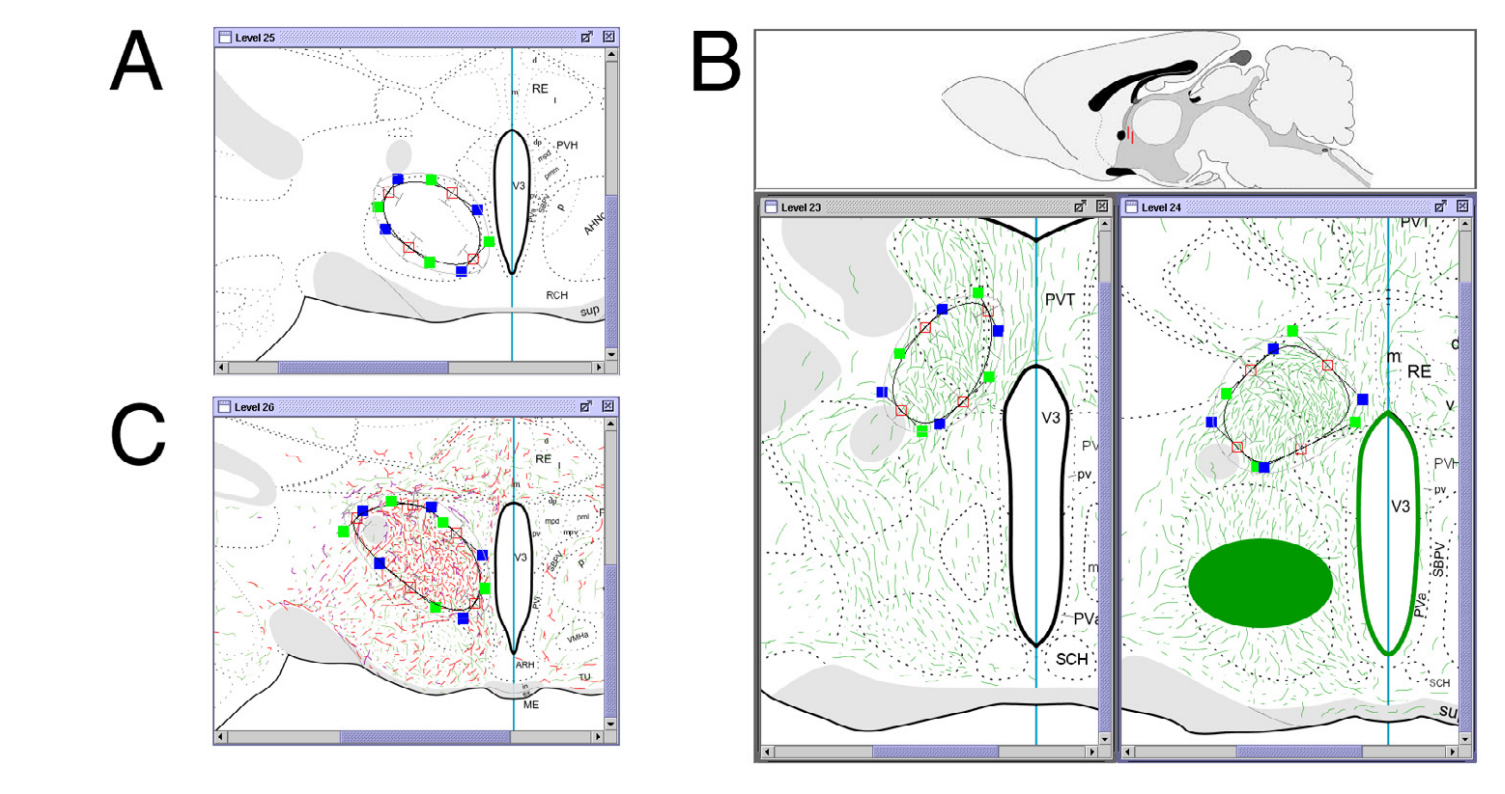
The brain-volume spatial primitive, used to delineate a putative terminal field in the hypothalamus (based on the example illustrated in Fig. 7). Chemoarchitectonic maps reprinted from Brain Research Reviews, vol 24, Risold, P.Y. and L.W. Swanson "Chemoarchitecture of the rat lateral septal complex". pp91-113, Copyright (1997), with permission from Elsevier. Maps of output connectivity of LS reprinted from Brain Research Reviews, vol 24, Risold, P.Y. and L.W. Swanson "Connections of the rat lateral septal complex". pp115-95, Copyright (1997), with permission from Elsevier. Maps of output connectivity of AHNc reproduced with permission of John Wiley & Sons, Inc. from Journal of Comparative Neurology, vol 348, Risold, P.Y. and L.W. Swanson "Organization of projections from the anterior hypothalamic nucleus: a Phaseolus vulgaris-leucoagglutinin study in the rat". Pages 1–40, Copyright ^© ^1994 Wiley Periodicals, Inc.

Brain-volumes are a tool available to the user for marking and describing brain regions of potential interest (Fig. 9). Essentially a brain-volume is a set of curves that combine to form a spatial primitive for designating related regions that may extend across atlas borders and/or atlas levels. Due to the lack of delineations in-between atlas levels, brain-volumes are not a true volumetric representation, but specify that delineated regions are related (and are assumed to be contiguous). A few examples better serve to illustrate the potential uses of the brain-volume. Continuing with the data from Figure 8, brain-volumes can be used to mark regions independently from atlas delineations at a number of distinct locations both within and across atlas levels (Fig. 9A). In Figure 9B, a useful illustration of this application would be to delineate and annotate the interesting result from Figure 8D. Thus it is possible to outline the part of the AHN that receives inputs from both LS injections and annotate it as 'dense inputs from LSr.m.v and LSr.vl.d (that both receive convergent DBH and AHN inputs)'. Figure 9C shows an example of a volume constructed to specify a terminal field generated by AHN projections. Brain-volumes are also independent of atlas delineations. Figure 9D illustrates an example where our region of interest not only disregards a border of an atlas structure, but a significant portion of it exists in an unnamed region of the brain. Brain-volumes and associated text annotations are saved in the underlying database, allowing both to be queried, retrieved and viewed along with all other available data.

## Discussion

NeuARt II provides an intuitive framework for browsing, comparing, interpreting and summarizing neuroanatomical data. This system capitalizes on the fact that virtually all neuroscience researchers use brain atlases to identify brain structures and that the electronic versions of the atlas plates can serve as templates to map anatomical data. Moreover, anatomical data mapped in this way is inherently in register with all other data mapped onto the same template.

The system in captures current methods of representing and comparing anatomical data. That is, the various maps are visually compared and salient points are noted. NeuARt II also offers additional functionality in two major ways: (A) data-maps in register can be superimposed and compared precisely in greater detail; (B) NeuARt II provides greater flexibility and variety in the way that data sets and atlas levels can be compared. Multiple windows can be opened, each capable of containing any atlas level and any number of overlaid drawings.

As illustrated in Fig. 8 and discussed above, data can be represented in sufficient detail to see small differences in patterns of neural projection or the distribution of peptide or gene expression that could signify a principle of organization at a significantly higher resolution than that of named structures (see [27,45]). The combination of enhanced precision allows neural circuitry to be explored in greater detail, which can potentially lead to formulation of testable hypotheses.

Surpassing the resolution implicit in the parcellation scheme of a given atlas is important and especially relevant because most bioinformatics knowledge bases concerned with neuroanatomical data have been nomenclature-driven [16,65,67-69]. There are several distinct limitations to this approach. Foremost is that for a structure to be indexed it must have a name. This is a non-trivial issue because unnamed areas do exist. These are predominantly the interstices between named structures, and can be quite large in places. A related aspect of this problem is that *how *a structure is indexed depends upon what it is called and there is no single accepted nomenclature for brain structures. Similarly, because two structures have the same name does not necessarily mean that they are homologous (refer to a place in the brain with the same size, shape and position), even in the same species. In and of themselves, these are not insurmountable problems. Advances are being made in ontological systems to catalogue, standardize, and reconcile differences in nomenclature and its referents (see [16,69,70]).

For anatomical data, the larger problem is that all attributes of a named structure must be indexed as if they apply equally to that structure as a whole. To the first approximation, this could be resolved by dividing areas into smaller and smaller parts, each with its own name. The success of such an approach presupposes that the brain can only be organized in one way meaning that alternatives would be formally reducible to one another. So far we have shown examples of three fundamentally distinct methods upon which delineation of brain structure can be based: cytoarchitectonics that uses the position, size, shape and packing density of the soma, hodology that defines groups based on their connections, and chemoarchitectonics that defines groups based on similarities in their neurochemistry. Each would suggest a parcellation scheme that differs significantly from the others in some way. Moreover, none of them are quantifiable in the way that would be needed to specify equivalence between them (and often the best approach is to combine them). In practical terms, advantages of maintaining a spatial representation can be seen easily in any map illustrated here. That is, few projections distribute equally to all parts of named structures and virtually all include information outside the domain of the named structures (*e.g*., see Fig. 9c).

It is important to note that the data-maps used by this application are not, nor are they intended to be, exact copies of the original data. Data mapped onto standard templates are by necessity interpretations of that data, carefully drawn by experts to represent the salient features of the data set in relation to defined atlas structures. The translation of experimental data onto atlas templates is performed with the aid of four sets of 'cues', which may be seen whilst examining brain tissue under a microscope.

The first set of cues is based on gross anatomical landmarks present in experimental tissue and is often illustrated in an atlas. A second set of cues arises from comparisons with a neutral standard, such as a 'Nissl' stain (which colors all cells). Generally speaking, it is standard histological practice to prepare a Nissl series (or the equivalent) in parallel with the experimental series for the express purpose of correlating with atlas structures. The third set of cues is derived by tracking a structure's incremental changes across sections to account for differences in the way its borders are drawn on adjacent atlas plates. Similarly, if a structure can be reliably identified in one section, the extent of nuclei that end between atlas levels can be determined by tracking incremental changes from section to section. Finally, the fourth set of cues is based on the structure's relationship with its neighbors. Considerable positional information can be gained by repeating the previous operations on the structures surrounding the area in question. This is especially useful if one of the neighbors is easy to identify and their borders coincide for a limited distance. This strategy is also helpful when issues arise from plane-of-section differences. We have argued that these cues must be taken into account in order to constrain and inform the translation process to allow an accurate and precise representation of a data set within the framework of a standard or reference atlas [5-7].

It is important to note, however, that this is not the only method of illustrating anatomical data. The camera lucida allows the experimenter to draw by hand what is seen under the microscope. One clear advantage of this method is that these drawings are more literal representations of the data, and relatively unbiased if sufficient area and landmarks are included. Localization is performed by drawing the area of interest onto another drawing of the equivalent area of the adjacent Nissl-stained section. Scaling and warping errors are introduced due to differential shrinkage produced by the two techniques and differential shear resulting from the tissue mounting process. Even so, these errors are relatively small and matching the two series is generally considered easy and accurate. Another issue is that the amount of tissue visible at the magnifications required to map data in detail is small and many contiguous drawings would be required to map an entire section. For this reason alone it is rarely the case that an experiment mapped with a camera lucida comprehensively shows the data of a whole section, much less an entire case. The time and effort required to map extensively may also account for the fact that brain landmarks and identified structures are often represented in insufficient detail to provide the context necessary to account for plane-of-section differences that invariably exist between experiments.

However, these are limitations of implementation entirely. There are two theoretical limitations that mapping onto standard templates addresses quite neatly. First, even if experimental data is exhaustively illustrated and unambiguously localized, one still is left with precise localization within an individual brain. The methods required to accurately transform experimental brains such that corresponding points are in register while the content and resolution of the data remains unchanged simply do not exist at this time (see [71]). Without formal methods of transformation and registry, comparison of data sets requires each researcher to mentally perform the same translation. Thus when comparing camera lucida drawings we find that we are performing the same operations as those required for mapping data onto a standard template, without the benefit of the five cues described above. By investing energy in performing this interpretation early in the process of mapping data, we remove the need to perform it every time a reader views a paper. The great advantage of the mapping approach is that all represented data are all registered in a single standard frame of reference and may therefore be compared easily and semi-quantitatively (for additional discussions of this topic, see [1,6,72]).

While offering an enhanced capability to compare atlas-mapped data qualitatively, the system is nevertheless implemented as a viewing (rather than an analysis) tool. In contrast, the field of spatial data analysis is a mature research area in the field of Geographical Information Sciences [73]. There are a number of computational approaches to cartographic data that could provide additional functionality. These include standardized statistical analysis software (*e.g*., see [74] for spatial components in the open-source statistics system 'R', [75]). One potential application could be to query the PHAL database for a summary of synaptic targets of a given injection. The candidate regions could theoretically be determined by capitalizing on the resolution afforded by vector based maps to assess the density branching fibers and the prevalence of terminal boutons – the two major characteristics of a terminal field. In the near term, we will first institute spatial queries based on the standardized components (conforming to the 'OpenGIS Geometry Model' [76]). This functionality shall operate by using the brain-volume template to delineate an area and then query the system for data falling within the region of interest. Future versions will also ultimately include the ability to compare data across atlases and the capability to embed into atlas plates additional types that are constrained, but not in complete register with an atlas. Examples of these data types include photographs, camera lucida drawings and other non-mapped data. A near term goal is to create an environment and appropriate tools with the capability to generate high quality graphical output to a standard at or above the standard for publication.

Clearly this project is primarily of great interest to neuroanatomists and other fields where different topographic maps need to be compared. It is also important to point out that NeuArt II is potentially relevant to all of neuroscience because neuroscientific data, independent of data type, must be associated with at least one location within brain tissue. Given that future programming work will involve developing NeuARt II to interoperate with other neuroanatomical informatics projects, we envision NeuArt as a gateway environment to manage, store and query all types of neuroscientific data (including bibliographic data) within an anatomical/spatial context.

As proponents of the process of mapping data into atlases, we feel that the impact of this work will be maximized if it provides an incentive for users to construct their own maps that then may be incorporated into a shared repository of maps for the community. As suggested by Figure 2, no single laboratory can generate all of the data necessary to describe the entire set of brain connections. A software project such as NeuARt II could provide the information infrastructure to address this problem *as a community*. Interested individuals would contribute any set of maps in register with an atlas on any scale – from entire data sets to small mapped sections of a single atlas plate. The accumulation of these small-scale partial maps would permit a global representation to be built up. Naturally, with this sort of approach, community-based issues such as quality control, intellectual property, and security would arise and would need to be dealt with by well-defined web-community software. At the very least, the Swanson laboratory will continue to publish neuroanatomical maps in journal articles. As these maps are published, it is straightforward to obtain permission from the publishers of the journal article to allow us to reproduce and disseminate the data for the published maps with NeuARt II. This could form part of the submission process of a general community based approach.

Although this project is primarily of interest to neuroanatomists, it is relevant to any field where topographic maps need to be compared (or where data of a specific type is linked to a spatial location). By its very nature, this qualification applies to most neuroscientific data, but could also apply to a much larger number of subjects in biomedicine. Given any histological atlas with data files (in an appropriate format), NeuARt II could provide a suitable informatics framework.

## Conclusion

Within this paper, we present 'NeuARt II', an informatics system that acts as a visual interface for experimental neuroanatomical work that is based on existing practices within the field. The system is free, open-source and is part of a general neuroinformatics knowledge-management environment (as well as having the capability to act as a standalone system). This system offers an opportunity for experimentalists to browse and view atlas-registered data more rapidly and easily than was previously possible by manual manipulation of large numbers of individual data files. The system also provides a foundation for contrasting two major methods of neuroanatomical mapping, one based on camera lucida techniques that presents each individual case as accurately as possible (without registration into an atlas) and the mapping techniques illustrated here that involve registering data into a standard atlas framework. The system can manage large numbers of atlas-registered neuroanatomical maps to allow users to navigate, compare and contrast data within these maps and make spatial annotations that may then be saved in the systems' underlying database. We envision this software becoming a platform for data-sharing across modalities within neuroscience.

## Availability and requirements

Project name: NeuARt II

Project home page: http://www.neuroscholar.org/neuart2.html

Operating system(s): Windows, Mac OS X, Linux

Programming language: Java

Other requirements: Java 1.4, MySQL

License: NeuroScholar license (essentially GNU GPL with added minor caveats).

Any restrictions to use by non-academics: none

## Abbreviations

AC anterior commissure; ACAv	anterior cingulate area, ventral part; ACB	nucleus accumbens; AHN	anterior hypothalamic nucleus; AHNa	anterior hypothalamic nucleus, anterior part; AHNc anterior hypothalamic nucleus, central part; AMd anteromedial nucleus of the thalamus, dorsal part; ARH arcuate nucleus of the hypothalamus; AVPV anteroventral periventricular nucleus, hypothalamuc; BST bed nuclei stria terminalis; CM central medial nucleus of the thalamus; COM periaqueductal gray, commisural nucleus; DMH dorsomedial nucleus of the hypothalamus; fx fornix IAD	interanterodorsal nucleus of the thalamus; IF interfascicular raphe nucleus; IG induseum griseum; LHAsfa lateral hypothalamic area, suprafornical region, anterior zone; LM lateral mammilliary nucleus; LPO lateral preoptic area; LS lateral septal nucleus; LSc lateral septal nucleus, caudal part; LSc.d lateral septal nucleus, caudal part, dorsal zone; LSc.d.d lateral septal nucleus, caudal part, dorsal zone, dorsal region; LSc.d.l lateral septal nucleus, caudal part, dorsal zone, lateral region; LSc.d.r lateral septal nucleus, caudal part, dorsal zone, rostral region; LSc.d.v lateral septal nucleus, caudal part, dorsal zone, ventral region; LSc.v lateral septal nucleus, caudal part, ventral zone; LSc.v.i lateral septal nucleus, caudal part, ventral zone, intermediate region; LSc.v.l lateral septal nucleus, caudal part, ventral zone, lateral region; LSc.v.m lateral septal nucleus, caudal part, ventral zone, medial region; LSr lateral septal nucleus, rostral part; LSr.dl lateral septal nucleus, rostral part, dorsolateral zone; LSr.dl.l lateral septal nucleus, rostral part, dorsolateral zone, lateral region; LSr.dl.m lateral septal nucleus, rostral part, dorsolateral zone, medial region; LSr.m lateral septal nucleus, rostral part, medial zone; LSr.m.d lateral septal nucleus, rostral part, medial zone, dorsal region; LSr.m.v lateral septal nucleus, rostral part, medial zone, ventral region; LSr.vl lateral septal nucleus, rostral part, ventrolateral zone; LSr.vl.d lateral septal nucleus, rostral part, ventrolateral zone, dorsal region; LSr.vl.v lateral septal nucleus, rostral part, ventrolateral zone, ventral region; LSv lateral septal nucleus, ventral part; MEPO median preoptic nucleus; MM medial mammillary nucleus, body; MPN medial preoptic nucleus; MS medial septal nucleus; NDB nucleus of the diagonal band; PAG periaqueductal gray; PH posterior hypothalamic nucleus; PMd dorsal premammilliary nucleus; PMv ventral premammilliary nucleus; PRC periaqueductal gray, precommisural nucleus; PSTN parasubthalamic nucleus; PT parataenial nucleus; PVH paraventricular nucleus of the hypothalamus; PVT paraventricular nucleus of the thalamus; RE nucleus reuniens; Rh rhomboid nucleus; RSPagl retrosplenial area, lateral agranular part; SCH suprachiasmatic nucleus; SF septofimbrial nucleus; SFO subfornical organ; SH septohippocampal nucleus; SUBv subiculum, ventral part; SUMl supramammilliary nucleus, lateral part; TU lateral hypothalamic area, tuberal nucleus; V3 third ventrical; VL lateral ventrical; VMH ventromedial nucleus of the hypothalamus.

## Authors' contributions

GAPCB, RHT and LWS wrote the paper. GAPCB and WCC designed and built the NeuARt and NeuroScholar systems. The implementation work was performed by WCC. RHT and LWS provided both the requirements specification of the software, and end-user feedback. They also made specific feature requests within the software development process. All authors have read and approved the final manuscript, except for WCC, who has left academia, but nonetheless contributed sufficiently to be an author on the final paper.

## Appendix A: the design of the database representation

**Figure 10:**
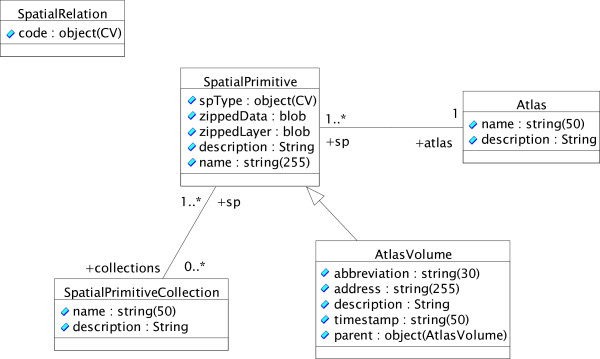
A UML class diagram describing the data format of the NeuARt II system.

Here we briefly describe the details of the underlying database representation used under the VPDMf for this application. The UML class diagram, shown below in Figure 10, illustrates the underlying representation used by the NeuroScholar platform database to store the views illustrated as a graph in Fig. 4. We present the schema as an object-oriented UML diagram, since the VPDMf system builder scripts automatically converts this representation to a relational database scheme. This is consistent with previous publications from our group on other representations constructed under the VPDMf. For more details of this transformation process, readers are referred to [63].

It is important to note that at this stage, we represent the internal structure of all graphical objects as SVG fragments that are stored as BLOB objects in the underlying MySQL database. In the future, if we wish to index these objects spatially, we will need to add appropriate tables where we unpack the structure of these spatial primitives. Not only will that greatly increase the size of data being stored, it may create concurrency issues as the spatial indices will reproduce the data contained in the SVG image files.

## Supplementary Material

Additional file 1 The data map from a PHAL injection into the rostral part of the lateral septal nucleus, ventrolateral zone, dorsal region (LSr.vl.d). A stack of SVG files that may be uploaded into the NeuARt system (see the file README.pdf for instructions) to demonstrate the system's viewing capabilities. Reprinted from Brain Research Reviews, vol 24, Risold, P.Y. and L.W. Swanson "Connections of the rat lateral septal complex". pp115-95, Copyright (1997), with permission from Elsevier. The data maybe downloaded as a ZIP archive which must then be expanded into a temporary directory. The archive file contains a README.pdf document which provides instructions for using the data in the file. Click here for file
