# Supplementary material for: The NeuARt II system: a viewing tool for neuroanatomical data based on published neuroanatomical atlases
Source: BMC Bioinformatics. 2006 Dec 13;7:531. doi: 10.1186/1471-2105-7-531 (PMC1770939; doi:10.1186/1471-2105-7-531)

NEUART II - INSTRUCTIONS FOR LOADING SAMPLE DATA FILE.

1. Run the application. You will see the following screen:

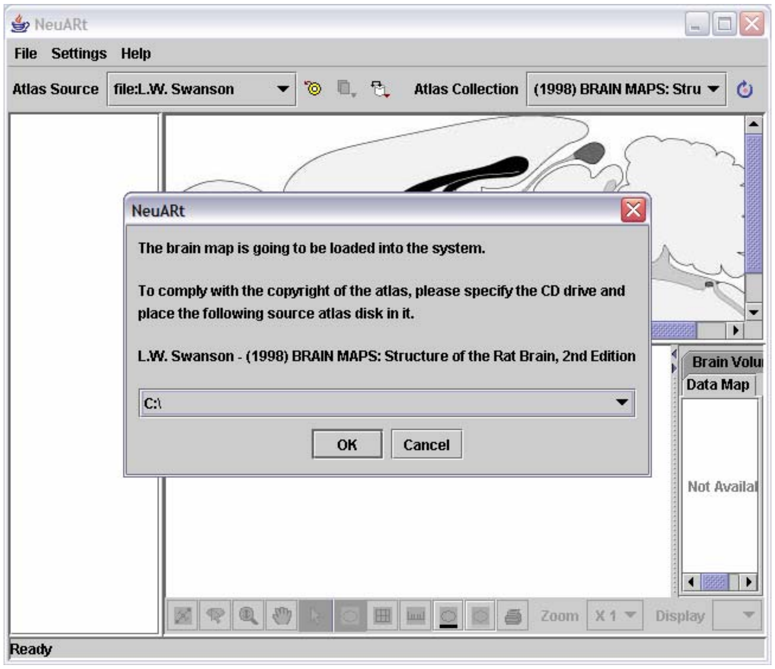

2. Place the atlas CD into the appropriate drive and select the drive number. Press OK. This runs the system and puts it into the ready state.

3. Click the button marked ‘ 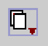 ’ in the tool bar and select ‘Import Data Map’. The following control will appear:

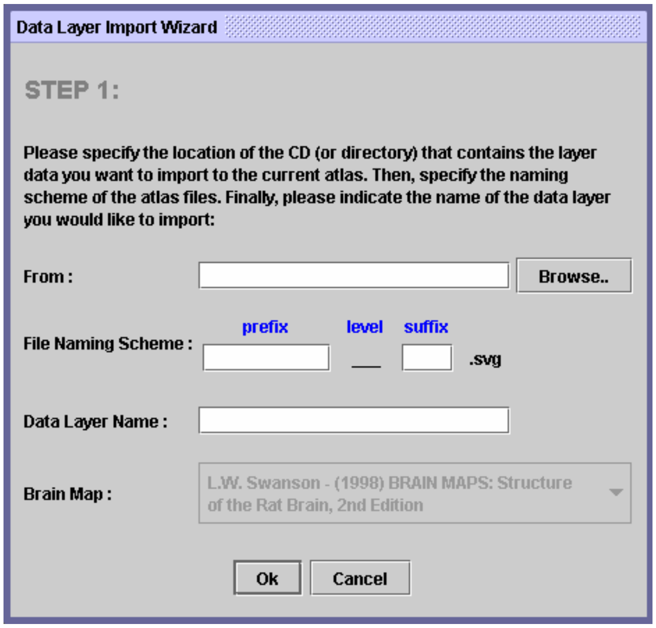

Within the box, click on the Browse… button and select the folder where you unzipped the data files to be uploaded.

Within the 'File Naming Scheme' box, type 'sample-' in the prefix box and leave the suffix box blank.

Within the 'Data Layer Name' box, type 'LSr.vl.d_PHAL' (note that the system requires that you type an underscore character for any spaces in the layer name).

Click OK.

4. The data will start to load and the following dialogue box will appear on the screen.

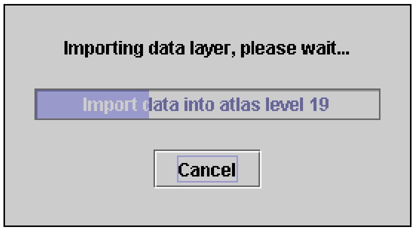

If the import process runs through all the levels of the atlas without loading data, you may have made a mistake entering data onto the form.

5. If the data loads, navigate to level 14 and you will see the following image.

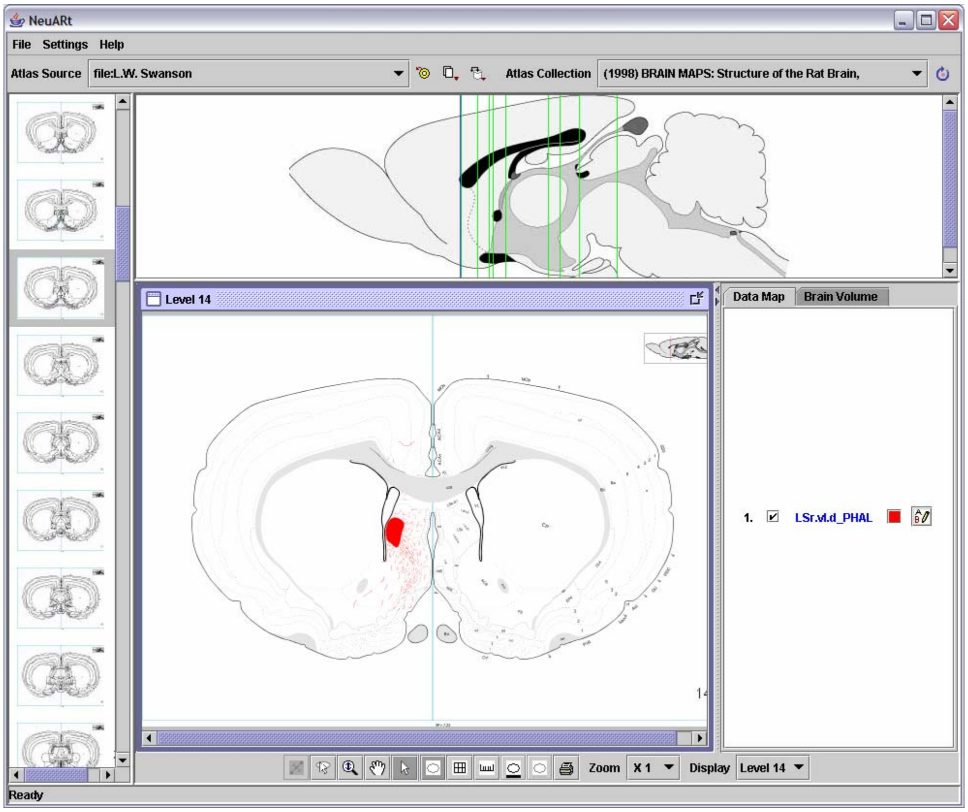

Supplement: Additional file 1 — The data map from a PHAL injection into the rostral part of the lateral septal nucleus, ventrolateral zone, dorsal region (LSr.vl.d). A stack of SVG files that may be uploaded into the NeuARt system (see the file README.pdf for instructions) to demonstrate the system's viewing capabilities. Reprinted from Brain Research Reviews, vol 24, Risold, P.Y. and L.W. Swanson "Connections of the rat lateral septal complex". pp115-95, Copyright (1997), with permission from Elsevier. The data maybe downloaded as a ZIP archive which must then be expanded into a temporary directory. The archive file contains a README.pdf document which provides instructions for using the data in the file. [file 1471-2105-7-531-S1.zip › README.pdf]
